# Effects of precipitation changes on soil bacterial community composition and diversity in the Junggar desert of Xinjiang, China

**DOI:** 10.7717/peerj.8433

**Published:** 2020-01-27

**Authors:** Ke Wu, Wenxuan Xu, Weikang Yang

**Affiliations:** 1 CAS Key Laboratory of Biogeography and Bioresource in Arid Land, Xinjiang Institute of Ecology and Geography, Urumqi, China; 2 Mori Wildlife Ecological Monitoring and Experimentation Station, Xinjiang Institute of Ecology and Geography, Chinese Academy of Sciences, Mori, China; 3 University of Chinese Academy of Sciences, Beijing, China

**Keywords:** Precipitation variation, Soil bacterial diversity, Drought, Junggar desert

## Abstract

Variation in precipitation can markedly affect the structure and function of soil microbial communities, especially in arid areas which are limited by water resources. Therefore, it is critical to understand how soil bacterial community composition and diversity will respond to variation in precipitation. In this study, we examined the soil bacterial community structure and diversity between five precipitation treatments (60% decrease, 30% decrease, control, 30% increase and 60% increase in precipitation) in the same arid site, in the Junggar desert of Xinjiang, northern China. The dominant bacterial phyla, present at similar frequencies in plots with different precipitation levels, were Actinobacteria, Proteobacteria, Bacteroidetes, Acidobacteria and Chloroflexi. The Shannon-Wiener and Chao1 indices of soil bacterial *α*-diversity were both positively correlated with plant diversity. Our results indicated that (1) extreme drought significantly decreased bacterial abundance and diversity compared with increased precipitation; (2) variation in precipitation did not change the dominant components of the bacterial communities; and (3) soil pH and total nitrogen concentration were the key factors affecting soil bacterial composition in the Junggar desert.

## Introduction

Global precipitation amounts and patterns will change in the context of global climate change (Cui, 2018). The changes in soil moisture caused by precipitation will directly affect water utilization by plants and soil microbes, and indirectly affect the functions and processes of terrestrial ecosystems. Soil microbes are one of the important groups of soil organisms, with almost all soil processes being directly or indirectly associated with soil microbes (Bardgett, 2005). The structure and diversity of soil microbial communities can reflect changes in processes affecting soil environmental quality, and are of great significance for maintaining soil quality and ecosystem stability (Witt & Setàlà, 2010). Bacteria are responsible for diverse metabolic functions that affect soil and plant health. All life forms rely on bacterial processes for their survival, and bacterial diversity is greater than the diversity of any other group of organisms (Kennedy, 1999).

Desert ecosystems are more sensitive than other regions to environmental changes, because of the characteristic stressful conditions of low water and nutrient availability (Makhalanyane et al., 2015). Moisture is believed to be one of the major constraints affecting microbial diversity, community structure and activity in arid ecosystems (Li et al., 2016). Variation in precipitation can affect soil bacterial communities directly by changing soil water availability (Bagayoko et al., 2000), and indirectly by altering soil nutrient availability (Xu et al., 2014) and plant community composition and productivity (Gray et al., 2011).

Over the past few decades, numerous studies have documented the response of soil bacterial communities to precipitation changes. However, as a result of extreme drought and temperature conditions, lack of soil organic matter, species-poor communities and community structure, and simple ecological functions in the desert, results from research into the effect of precipitation change on soil bacterial community in the desert are inconsistent. Most studies have shown that changes in precipitation can affect soil bacterial diversity and community composition (Ochoa-Hueso et al., 2018; She et al., 2018). For example, Zhang et al. (2018) found that the relative abundances of Proteobacteria and Bacteroidetes did not change significantly with the addition of water, but that the frequency of Acidobacteria clearly decreased in response to water addition, whereas the relative abundance of Planctomycetes increased first and then decreased under two different water addition regimes. On the contrary, other studies have indicated that variation in precipitation did not change the diversity and the composition of the dominant bacterial communities (Bachar et al., 2010; Wang et al., 2014; Zhang et al., 2019). Collectively, the inconsistent responses of microbes to precipitation changes may result from ecosystem-specific responses, historical precipitation regimes, or indirect water-induced environmental factors (She et al., 2018).

Plant community composition and diversity is likely to affect the diversity of microbial species (Johnson et al., 2003). Some research has shown that an increase in plant diversity can increase the bacterial community diversity (Stephan, Meyer & Schmid, 2000; Loranger-Merciris et al., 2006), because the diversity of bacteria is regulated by the composition of growth-limiting resources, which are mainly based on soil organic matter (Lavelle et al., 1995). However, some studies have shown inconsistent results. For instance, Kuramae et al. (2011) indicated that no significant changes in bacterial community structure and diversity occurred in response to an increase in plant diversity. Whether there is a relationship between plant diversity and soil bacterial diversity depends on the type of biological interactions between them and the temporal and spatial scale of organisms affected by ecological factors (Hooper et al., 2000).

The area of temperate desert in Xinjiang in northern China accounts for about 53% of the total desert area in China (Cui, 2018). Most researchers thought that the rainfall in Xinjiang tended to increase (Qin, Ding & Su, 2006; Ding et al., 2007), but some researchers suggested that Xinjiang will enter a long-term period of low precipitation in the future (Ren et al., 2015). Changes in precipitation may alter the activities of soil microbes and thus, affect the biogeochemical cycles in terrestrial ecosystems (Zhang et al., 2013). Therefore, an accurate understanding of the response of soil microbes to precipitation changes will help us accurately predict how ecosystem functions will change in response to future climate conditions. The soil microbial community composition is influenced by a wide variety of factors, including soil type and texture, moisture, pH and temperature (Fierer & Jackson, 2006). Many of these factors interact with one another and have both direct and indirect effects on the soil microbial community (Buyer et al., 2010). Identification of the factors that influence microbial community composition in the desert will have significant impact on our understanding of how precipitation change will affect soil bacterial community.

We took advantage of an on-going precipitation manipulation experiment in the Junggar desert of Xinjiang to investigate how increases and decreases in precipitation might alter soil bacterial diversity and bacterial community composition. Our main goals were (1) to test how soil bacterial diversity and community composition respond to increased precipitation and extreme drought; (2) to determine the relationship between plant diversity and bacterial diversity; and (3) to identify the key factors that determine soil bacterial community structure. The results from this current study should advance a potential mechanistic understanding of the responses of desert microbial communities to global environmental changes.

## Methods

### Study site

**Figure 1:**
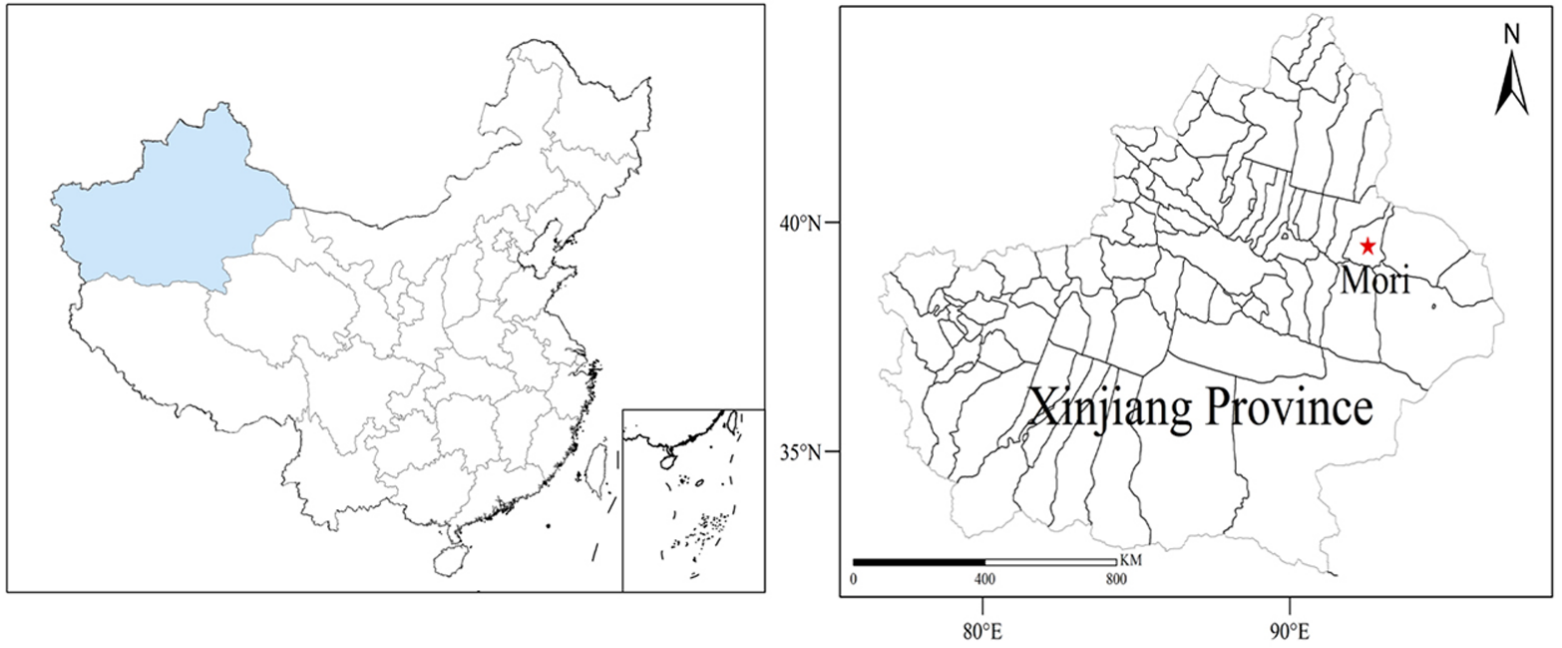
Experimental site.

The study was conducted in a desert site at the southeastern edge of the Junggar basin at Mori County, Xinjiang, China. The study area (43°59′N, 90°48′E) lies on the alluvial fan of the Tianshan Mountains, with an elevation ranging from 1,000 to 1,200 m above sea level (Fig. 1). The major soil type is the desert gray soil that is highly susceptible to wind erosion. The soil is highly alkaline (pH = 9.08 ± 0.05) with low fertility. The climate of this area is arid and cold (+20 °C on average in July, and −15 °C on average in January). The mean annual precipitation is 150 mm, falling mainly in the spring and winter. The mean soil contentration of total nitrogen and organic matter were 0.49 g kg^−1^ and 6.13 g kg^−1^, respectively.

The rainfall in the study area is concentrated mainly in the spring, from the beginning of April to the end of May, which is the period of the most vigorous growth of the ephemeral plants and also of the greatest species richness (Xu et al., 2016).

The experimental plots, where the present study was conducted, were fenced as exclosures in 2014. The study area is typical desert, dominated by the shrub Seriphidium borotalense. The vegetation is typically sparse (∼25% coverage) and short (∼20 cm), with an admixture of scattered shrubs dominated by *Haloxylon ammodendron, Atraphaxis spinosa,* and *Reaumuria songarica,* among others. The herbaceous vegetation in the area flourishes in May and June. The most common herbaceous plants are *Ceratocarpus arenarius, Salsola paulsenii, Halogeton glomeratus, and strigosella (Malcolmia) Africana*.

### Experimental design

We analyzed the precipitation data in Mori County from 1960 to 2008 (data being obtained from local meteorological stations) and determined that the mean annual precipitation ranged from −30% to +30% of the average. Furthermore, precipitation is predicted to increase by 30% in northern China over the next 30 years (Liu et al., 2017), so the water addition treatments were adjusted by an extra +30% precipitation or by an extra +60% precipitation to represent an extreme situation; we also reduced precipitation by 30% and 60% to represent drought and extreme drought treatments, respectively.

In April 2016, fifteen plots were established, with each of them being 2 m × 2 m, with 3 m buffer zones between any two adjacent plots to avoid any ‘edge effect’. The fifteen plots were repreasented three replicates for each of five levels of precipitation: 60% decreased precipitation (−60%), 30% decreased precipitation (−30%), control (CK), 30% increased (+30%), and 60% increased precipitation (+60%).

**Figure 2:**
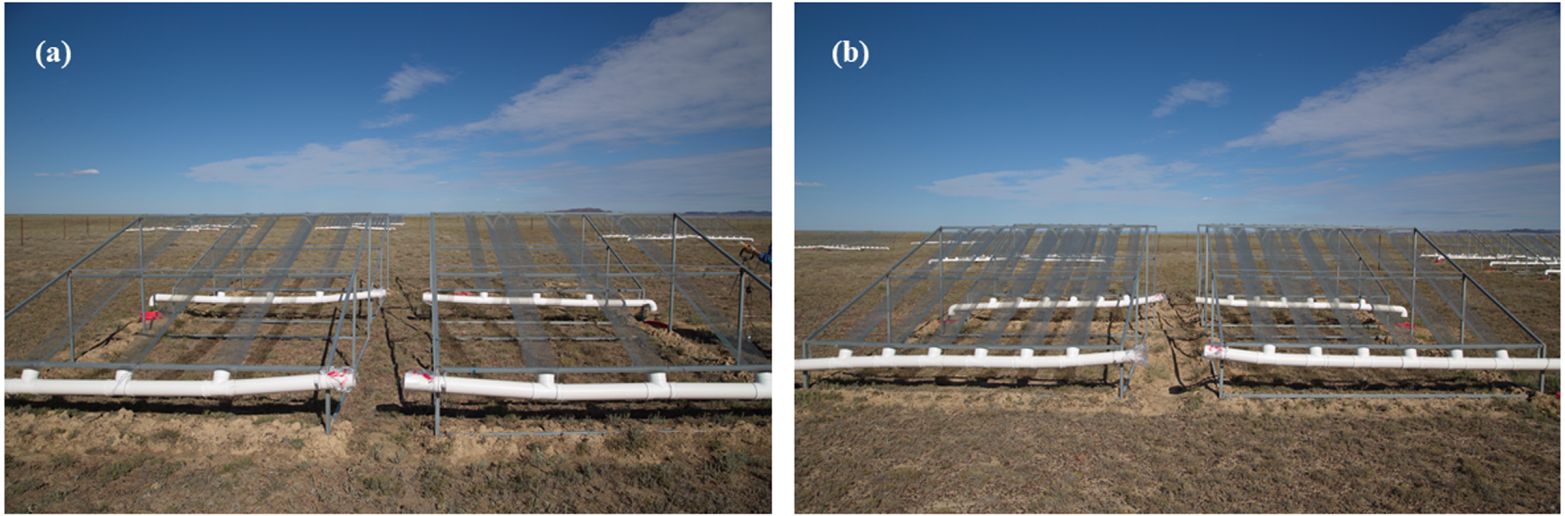
Precipitation decreased by 30 percent (A) and 60 percent (B).

For the drought treatments, rainout shelters had a metal frame supporting V-shaped clear acrylic bands, without a UV filter, covering an area of 9 m^2^ (3 m × 3 m). The roof had a 20° inclination, and on the lowest side, it had a gutter that channeled any intercepted water into a flexible storage tank, with a capacity of 170 L, made of white canvas covered with PVC (Knapp et al., 2017). The roof suspended over treatment plots only minimally affected other key environmental variables, and this approach is widely used in studies of many types of ecosystems (Yahdjian & Sala, 2002). Increased precipitation treatment plots received ambient precipitation collected from the roofs over the drought treatment plots immediately after rainfall. The ambient precipitation plots at each site served as controls (Fig. 2).

The number of plant species, percentage cover and plant height were determined in each sample plot in May 2018 (Table 1).

**Table 1:** The general vegetation of the study site.

Variable	Annuals	Perennials	Shrubs
Number of species	14.00 ± 1.26^a^	2.00 ± 0.05	5.00 ± 0.61
Mean cover (%)	10.03 ± 0.04	5.14 ± 0.01	24.91 ± 0.25
Mean height (cm)	2.80 ± 0.25	14.20 ± 0.01	7.53 ± 1.37

**Notes.**

a mean ± standard error (SE).

### Calculation of plant community diversity

Simpson’s diversity index, richness index, Shannon-Wiener index and Pielou evenness index were used to measure community diversity (Lloyd & Ghelardi, 1964; Ma, 1994).

\begin{eqnarray*}& & \mathrm{Richness~ index(R)}:\mathrm{R}=\mathrm{S} \end{eqnarray*}

\begin{eqnarray*}& & {\mathrm{H}}^{{^{\prime}}}=-\sum _{i}^{S} \left( Pi \right) (\ln \nolimits  Pi) \end{eqnarray*}

\begin{eqnarray*}& & \mathrm{Pielou~ index(E)}:E={H}^{{^{\prime}}}/lnS \end{eqnarray*}

Where S is the number of species, and Pi is the proportion of individuals belonging to i species.

### Soil sampling and analyses

Soil samples were carefully collected from each plot at the topsoil level (0–10 cm) in early August 2018 using an auger (3.5 cm diameter). The diagonal sampling method was adopted to set up four sampling points in each plot, with all soil samples from each replicate plot being mixed uniformly and passed through a 2.0 mm sieve. The fresh soil sample was divided into two subsamples, of which one was air-dried and used to measure physical and chemical properties and the other was stored at −80 °C prior to DNA extraction (Petrosino et al., 2009).

Soil water content was determined by taking soil cores in each plot and then oven-drying the cores for 48 h at 105 °C. Soil pH was measured in a soil water suspension (1:2.5 w: v) with a digital pH meter (SevenEasy, METLER TOLEDO Corporation). Soil organic matter (SOM, g kg^−1^) was determined by oxidation with dichromate in the presence of H_2_SO_4_ without application of external heat, as described by Rowell (1994). Total nitrogen (TN, g kg^−1^) was determined by the Kjeldahl method (8420, FOSS Analytical Corporation, Denmark); total phosphorus (TP, g kg^−1^) was determined using the ignition method of Saunders & Williams (1955) with P detection by acid-molybdate colorimetry (Cary60, Agilent Technologies Inc.); and total K (TK, g kg^−1^) was determined by Na_2_CO_3_ extraction (Olsen & Sommers, 1982) and measurement with an atomic absorption spectrometer (IRIS Advantage-ER, Thermo Jarrell Ash Corporation). Available N (NH${}_{4}^{+}$ and NO${}_{3}^{-}$) was extracted using KCl and determined calorimetrically in the soil extracts (8420, FOSS Analytical Corporation, Denmark). Available P (AP) was determined using the Kelowna method as described by Van Lierop (1988), using a solid: liquid extraction ratio of 1:5 and quantified by spectrophotometry (Cary60, Agilent Technologies Inc.). Available K (AK) were extracted with the Mehlich III solution (John et al., 2007) and measured using an inductively coupled plasma atomic-emission spectrometer (IRIS Advantage-ER, Thermo Jarrell Ash Corporation).

### Soil DNA extraction and amplification of 16S rRNA genes

Extraction and purification of the total genomic DNA from soil was achieved using the Fast- DNA SPIN Kit for Soil (MP Biomedicals). The quality of the extracted genomic DNA was visualized by electrophoresis through a 1% agarose gel, and the DNA was stored at −80 °C until use (Caporaso et al., 2010). We amplified a region of the 16S rRNA gene for bacteria. The primers used for sequencing were 341F (CCTAYGGGRBGCASCAG)/806R (GGACTACNNGGGTATCTAAT) flanking the V3 and V4 regions of the 16S rRNA gene (Berg et al., 2012). All PCR reactions were carried out in 30 µL reactions with 15 µL of Phusion® High-Fidelity PCR Master Mix (New England Biolabs); 0.2 µM of forward and reverse primers, and 10 ng template DNA. Thermal cycling consisted of initial denaturation at 98 °C for 1 min, followed by 30 cycles of denaturation at 98 °C for 10 s, annealing at 50 °C for 30 s, and elongation at 72 °C for 30 s, with a final incubation at 72 °C for 5 min. The same volume of 1 × loading buffer (contained SYB green) and the PCR products was subjected to electrophoresis on 2% agarose gel for detection. PCR products were mixed in equidensity ratios. Then, mixture PCR products was purified with GeneJETTM Gel Extraction Kit (Thermo Scientific). Samples with bright main strip between 400–450 bp were chosen for further experiments. PCR products was mixed in equimolar ratios. Then, mixture PCR products was purified with GeneJET Gel Extraction Kit (Thermo Scientific). The purified PCR amplicons products were sequenced on an Illumina MiSeq platform (Illumina Inc., San Diego, CA, USA) at Novogene Bioinformatics Technology Co. Ltd. (Beijing, China).

### Sequence bioinformatics analysis

Firstly, using Cutadapt (v1.9.1, http://cutadapt.readthedocs.io/en/stable/) was used to shear reads low quality part, then, the data from each sample was split from the obtained reads based on Barcode, and raw reads were obtained by cutting off Barcode and primer sequence for preliminary quality control. Secondly, raw reads were compared with the species annotation database through UCHIME. Algorithm to detect chimeric sequences and finally to remove the chimeric sequences, so as to obtain the clean reads. Clustering analysis was performed using UPARSE software (version 7.0.1001, http://drive5.com/uparse/). Sequences with ≥97% similarity were assigned to the same operational taxonomic units (OTUs). We got 3,207 OTUs totally and we selected a representative sequence for each OTU and use the RDP classifier to annotate taxonomic information for each representative sequence. In order to compute alpha-diversity, we rarefied the OTU table and calculate three metrics: Chao1 estimates the species abundance; Observed Species estimates the number of unique OTUs found in each sample, and the Shannon-Wiener index estimates the abundance and evenness of the species present. Rarefaction curves were generated based on these three metrics.

Species taxa analysis was conducted using the Mothur method and the small subunit ribosomal RNA (SSU rRNA) database (Wang et al., 2007) of SILVA132 (http://www.arb-silva.de/) (threshold value was 0.8∼1).

### Bacterial community diversity

The data of each sample were normalized, where the least amount of data in the sample was taken as the standard for normalization, and the subsequent alpha-diversity analyses were based on data following homogenization. QIIME software (version 1.9.1, http://qiime.org/) was used to calculate the diversity values of the samples, including Chao1 index, Shannon-Wiener index, and Simpson’s diversity index. R software (Version 2.15.3, http://www.r-project.org/) was used to analyze the differences between the groups of alpha-diversity indexes and to calculate a rarefaction curve (Edgar, 2013). Species richness and diversity of the bacterial community were characterized by the Chao1 and Shannon-Wiener indexes, respectively and the sequencing depth index was taken as the measure of sequencing coverage (Wang et al., 2015).

Chao1 index (Chao, 1984):

\begin{eqnarray*}\text{Chao}1={S}_{obs}+ \frac{{F}_{1}({F}_{1}-1)}{2({F}_{2}+1)} \end{eqnarray*}

Where S_obs_ is the number of observed OTUs, F_1_ is the number of OTUs with only one sequence, and F_2_ is the number of OTUs with only two sequences.

Simpson’s diversity index (Magurran, 2014):

\begin{eqnarray*}C=1-\sum P_{i}^{2} \end{eqnarray*}

Where P_*i*_ is the proportion of the community represented by OTU *i*.

### Rarefaction curve

**Figure 3:**
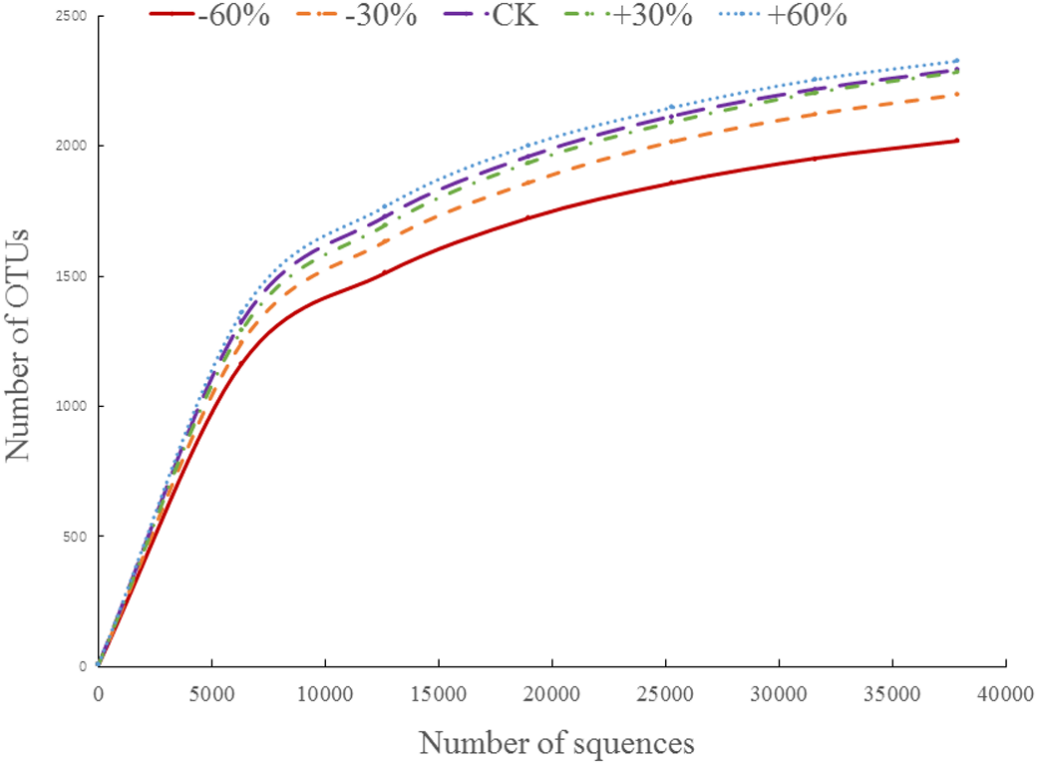
The rarefaction curve.

Rarefaction curves can be used to understand the depth of sampling of a community, compared with its total diversity, and can reflect the rationality of sequencing data volume (Olszewski, 2004). The rarefaction curve showed a flat state, but one which had not reached a saturated state, indicating that sampling was reasonable and that the result represented the bacterial community structure in the soil environment (Fig. 3).

### Statistical analysis

All data were expressed as the mean ± standard error (SE) of the mean. All data for a particular variable were first checked for normal distribution prior to analysis by using the Kolmogorov–Smirnov Z test. When the data were confirmed to be normally distributed, a parametic one-way measures analysis of variance (ANOVA) and multiple pairwise comparison analyse (Least Significant Difference, LSD) was carried out with SPSS 20.0 (IBM, Armonk, NY, USA), to measure the differences in soil properties and plant diversity among the five treatments. When the data were not normally distributed, the non-parametric Kruskal-Wallis test was used. The threshold probability level for significance in both cases was *P*<0.05. A significance test was conducted with the pair-wise PERMANOVA analysis method using the ‘adonis’ function in vegan package of R software (version 2.15.3, http://www.r-project.org/), based on the Bray-Curtis distance measure, to analyze differences in microbial community diversity. We used linear regression and Pearson’s correlation analysis to study the relationship between plant and bacterial diversity. Redundancy analysis (RDA) of the soil bacterial community was analyzed by CANOCO 4.5 (Microcomputer Power, Ithaca, NY, USA) to analyze the influence of environmental variables on the composition of the soil bacterial community. Structural equation modeling (SEM) was used to obtain a mechanistic understanding of how soil properties and plant diversity mediated changes in soil bacterial diversity under different precipitation regimes, by AMOS 17.0 (IBM, Armonk, NY, USA). SEM analysis was performed with the specification of a conceptual model of hypothetical relationships, assuming that precipitation altered soil pH, soil water content and plant diversity, each of which, in turn, affected bacterial community diversity (Zeng et al., 2016). In SEM analysis, we compared the model-derived variance–covariance matrix against the observed variance–covariance matrix, and data were fitted to the models, using the maximum likelihood estimation. Adequate model fits were indicated by the *χ*^2^ test (df > 5; *P*>0.05) and a low root mean square error of approximation (RMSEA) (*P* < 0.05). The final model was improved by removing relationships between observed variables from prior models, based on these indices.

## Results

### Soil properties and plant diversity index

After 2.5 years of the precipitation treatments, the physicochemical parameters of soil in the 15 plots were determined. The soil properties are summarized in Table 2. The pH values were similar (averaging 9.38 ± 0.14) in all plots. Soil water content (SWC) ranged from 4.56% to 6.01% across the plots, with soil pH and SWC being highest in the +30% plots. Compared with the control (CK), +30% and +60% plots, decreasing precipitation by 60% (−60%) significantly reduced the soil organic matter content (SOM), and there was a significantly lower SOM in −30% than in +60% (Table 2). The soil water content (SWC) under the −60% treatment was significantly lower than under the other four treatments, but any differences in SWC between the other four treatments were not significant. There was a general trend of increasing SOM, TN and SWC with increasing precipitation.

**Table 2:** Physical and chemical properties of topsoil under different precipitation regimes.

Variable	Precipitation regime				
	−60%	−30%	CK	+30%	+60%
pH	9.19 ± 0.08a	9.44 ± 0.16a	9.24 ± 0.09a	9.56 ± 0.03a	9.45 ± 0.24a
SOM	4.56 ± 0.12c	5.00 ± 0.49bc	5.25 ± 0.20ab	5.29 ± 0.14ab	6.01 ± 0.10a
SWC (%)	2.20 ± 0.01b	3.03 ± 0.01a	3.25 ± 0.01a	4.29 ± 0.01a	4.08 ± 0.01a
TN	0.22 ± 0.03a	0.23 ± 0.05a	0.25 ± 0.05a	0.26 ± 0.06a	0.27 ± 0.06a
TP	0.56 ± 0.01a	0.56 ± 0.02a	0.53 ± 0.02a	0.54 ± 0.02a	0.55 ± 0.04a
TK	19.04 ± 0.86a	19.80 ± 0.44a	19.69 ± 0.48a	19.05 ± 0.17a	19.57 ± 0.26a
AN	13.23 ± 2.40a	13.49 ± 2.74a	15.36 ± 2.79a	15.98 ± 2.37a	13.5 ± 3.84a
AP	8.14 ± 0.32a	7.92 ± 0.83ab	8.17 ± 0.40a	6.19 ± 1.46b	7.43 ± 1.01ab
AK	241.40 ± 20.14a	269.50 ± 36.20a	210.90 ± 11.53a	247.80 ± 41.66a	193.10 ± 31.52a

**Notes.**

Data are presented as mean ± SE. CK, control (ambient precipitation). For a particular variable, any two samples with a common letter are not significantly different (*P* > 0.05).

SOM (g kg^−1^) soil organic matter SWC soil water content TN (g kg^−1^) total nitrogen TP (g kg^−1^) total phosphorus TK (g kg^−1^) total potassium AN (mg kg^−1^) available nitrogen AP (mg kg^−1^) available phosphorus AK (mg kg^−1^) available potassium

The plant species richness ranged from 5.80 to 8.00 across the plots, being highest in the +60% plot. The Shannon-Wiener index varied from 1.23 to 1.69, and the Pielou and Simpson indices were in the range 0.63–0.82 and 0.23–0.43, respectively (Table 3), with no significant differences in any of these variables between the treatments.

**Table 3:** Physical and chemical properties of topsoil under different recipitation regimes. Data are presented as mean ± SE. CK, control (ambient precipitation). For a particular variable, any two samples with a common letter are not significantly different (*P* > 0.05).

	Precipitation regime				
	−60%	−30%	CK	+30%	+60%
Richness	5.70 ± 0.72a	7.30 ± 0.27a	6.60 ± 0.27a	7.00 ± 0.47a	8.00 ± 0.82a
Shannon–Wiener index	1.23 ± 0.03a	1.42 ± 0.02a	1.40 ± 0.02a	1.45 ± 0.06a	1.69 ± 0.09a
Pielou index	0.70 ± 0.05a	0.82 ± 0.04a	0.63 ± 0.04a	0.81 ± 0.07a	0.80 ± 0.07a
Simpson index	0.39 ± 0.04a	0.43 ± 0.04a	0.29 ± 0.00a	0.30 ± 0.02a	0.23 ± 0.05a

### Soil bacterial diversity

A total of 392,089 optimized sequences were obtained. The number of optimized sequences from each soil sample over the different precipitation regimes ranged from 75,777 to 80,493, with a mean ± SE of 78,418 (±892.61) sequences per sample. Soil bacterial diversity and richness under the extreme drought treatment (−60%) were significantly lower than under treatments with higher precipitation. The bacterial diversity and richness both revealed responses to precipitation in the order: +60%>CK>+30%>−30%>−60% (Tables 4 and 5).

**Table 4:** Variance analysis of alpha-diversity and richness of soil bacterial communities.

Diversity index	*df*	*F*	*P*
Chao1	4	2.10	**0.026**
Shannon index	4	3.432	**0.042**
Simpson index	4	3.224	0.061

**Table 5:** Alpha-diversity and richness of soil bacterial communities under the different precipitation treatments. Data are presented as mean ± SE. CK, control (ambient precipitation). For a particular variable, any two samples with a common letter are not significantly different (*P* > 0.05).

Diversity index	Precipitation regime				
	−60%	−30%	CK	+30%	+60%
Chao1	2156.47 ± 75.81b	2390.46 ± 96.93ab	2471.93 ± 75.87ab	2463.31 ± 79.83a	2503.69 ± 74.88a
Shannon index	8.82 ± 0.13b	9.09 ± 0.09ab	9.30 ± 0.08ab	9.21 ± 0.13a	9.42 ± 0.06a
Simpson index	0.991 ± 0.001a	0.994 ± 0.001a	0.995 ± 0.000a	0.994 ± 0.001a	0.996 ± 0.000a

**Figure 4:**
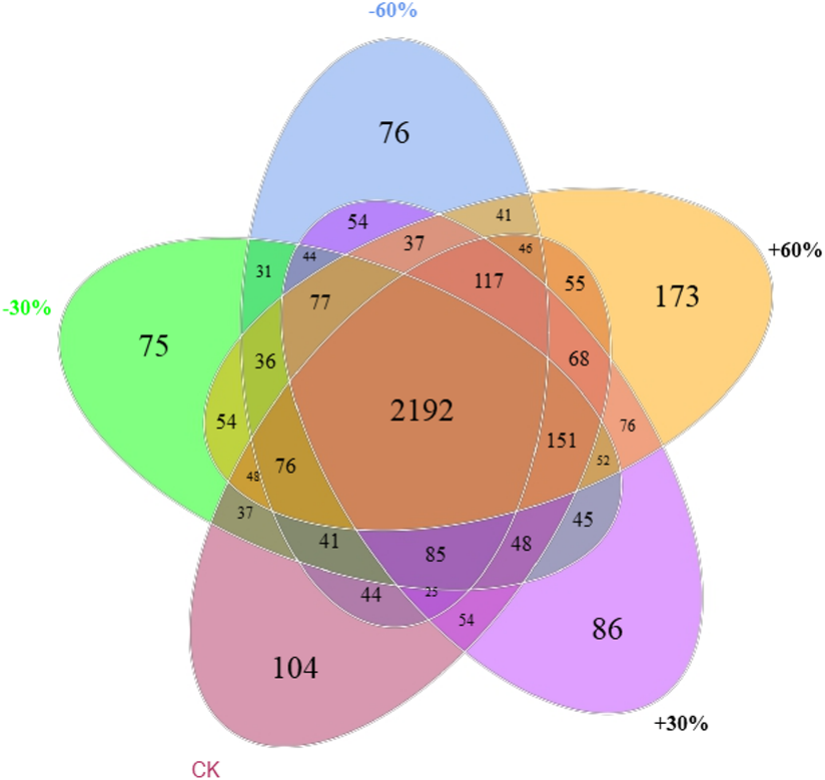
Venn diagram.

Figure 4 depicts a Venn diagram representing the unique and overlapping OTUs under the five treatments, indicating that most of the members comprising each of the soil communities were similar. The number of unique OTUs decreased with decreasing precipitation, and was significantly higher in the +60% precipitation plots than in the −60% and −30% plots (Fig. 4).

### Relationship between bacterial diversity and plant diversity

Bacterial diversity was positively correlated with plant diversity. The Shannon-Wiener index and the Chao1 index of soil bacterial diversity were both positively correlated with plant diversity, but the relationship was significant only in the +30% plot for the Shannon-Wiener index and was significant in the −30% and +30% plots for the Chao1 index (Table 6). Bacterial richness was positively correlated with plant richness. There were significant differences in different treatments, except for the −30% plot (Table 7).

**Table 6:** The correlation between bacterial diversity and plant diversity.

Plant Shannon index	Bacteria Shannon index		Bacteria Chao1 index	
	r	*P*	r	*P*
−60%	0.38	0.32	0.78	0.12
−30%	0.79	0.11	0.92	**0.02**
CK	0.63	0.25	0.75	0.14
+30%	0.94	**0.02**	0.98	**<0.01**
+60%	0.57	0.32	0.42	0.66

**Table 7:** The correlation between bacterial richness and plant richness.

Plant Richness	Bacteria Richness	
	r	*P*
−60%	0.94	**0.02**
−30%	0.75	0.15
CK	0.92	**0.03**
+30%	0.92	**0.03**
+60%	0.96	**0.01**

**Figure 5:**
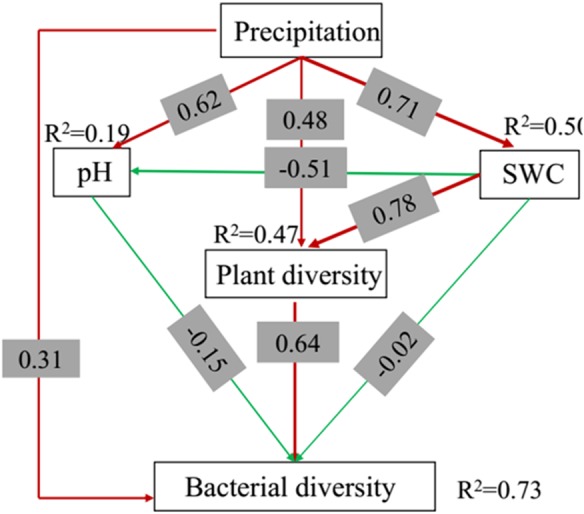
Structural equation model (SEM) of the effects of the precipitation on the bacterial diversity in the top soil (0–10 cm) soil depth.

The response of bacterial diversity to changes in precipitation was investigated using the SEM model. We compared multiple candidate models of SEM and selected a best model (*χ*^2^ = 0.300, *P* = 0.584, RMSEA = 0.00), which accounted for 19% and 50% of the variation in pH and SWC, respectively, 47% in plant diversity, and 73% in bacterial diversity. Increased precipitation was associated with increased bacterial diversity both directly and indirectly, via increased soil pH, SWC and plant diversity. Changes in plant diversity resulted in associated shifts in bacterial diversity (Fig. 5). The relationships between the other variables were not significant, but they clearly improved the model when incorporated together.

### Bacterial community structure

**Figure 6:**
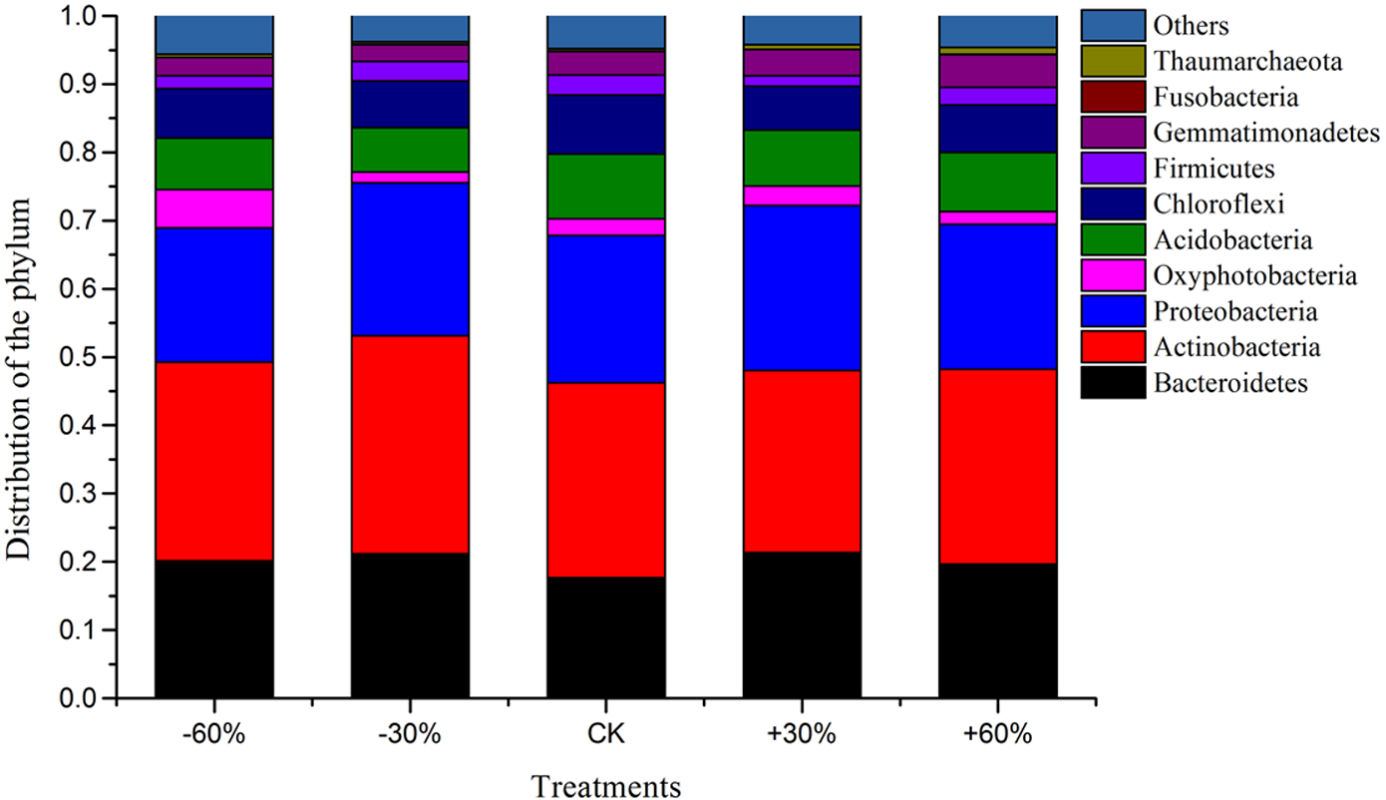
Bacterial composition of the different communities.

Soil bacterial community composition was consistent among the different precipitation treatments (Fig. 6). The bacterial communities from each treatment consisted of eight phyla, which were as follows: Actinobacteria (29% of the total amount of reads), Proteobacteria (22%), Bacteroidetes (20%), Acidobacteria (8%), Chloroflexi (7%), Gemmatimonadetes (3.47%), Oxyphotobacteria (2.89%) and Firmicutes (2.35%). The response in frequency of all phyla to variation in precipitation was inconsistent. The abundance of Gemmatimonadetes increased with increased precipitation, but the difference was not significant. The abundance of Proteobacteria and Bacteroidetes peaked at the +30% precipitation treatment, but there was no obvious trend with respect to how the abundance of the other groups in the bacterial community varied in response to precipitation. There was no significant difference in community composition among the treatments (*P* > 0.05) (Table 8).

**Table 8:** Comparison between bacterial community composition at different precipitation regimes based on pairwise PERMANOVA. *F* represents the test value of *F*; *R*^2^ represents the ratio of group variance to total variance; *P* < 0.05.

Group comparison	*F*	*R*^2^	*P*
−60%–−30%	0.715	0.152	0.800
−60%–CK	0.701	0.149	0.900
−60%–+30%	0.825	0.171	0.700
−60%–+60%	0.736	0.155	0.801
−30%–CK	0.876	0.180	0.701
−30%–+30%	0.793	0.165	0.801
−30%–+60%	0.902	0.184	0.701
CK–+30%	1.100	0.216	0.200
CK–+60%	0.694	0.148	0.900
+30%–+60%	0.821	0.170	0.701

### Relationships between soil properties and soil bacterial communities

**Figure 7:**
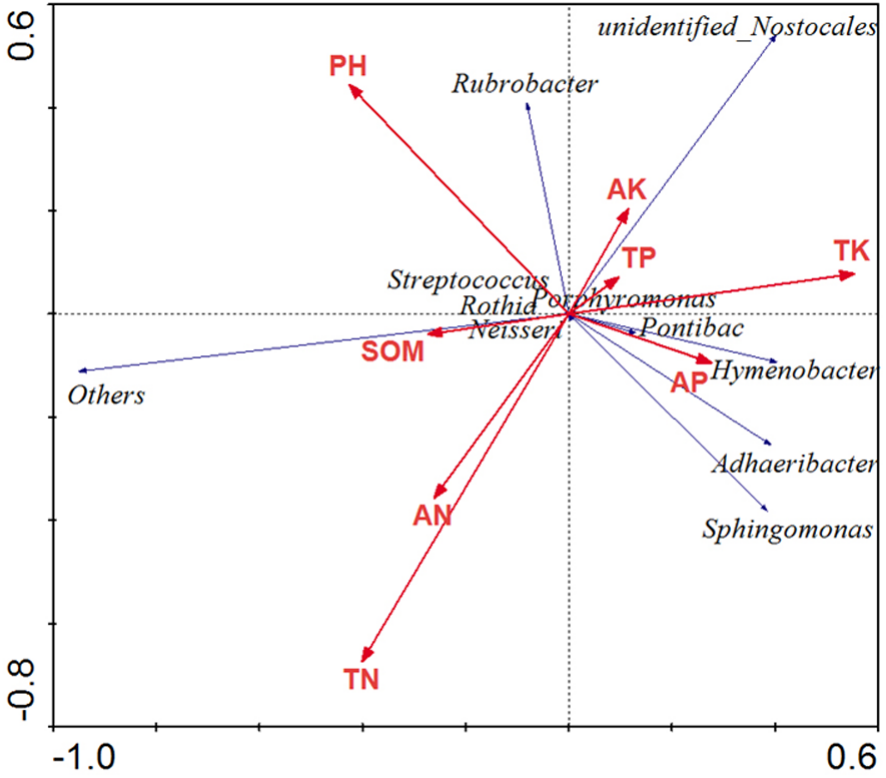
Redundancy analysis to explore relationship between microorganism community (genus) and physiochemical characteristic.

The results of the RDA showed that the first two axes accounted for a total of 59.6% of the variance in bacterial community changes, with the first axis accounting for 41.2% and the second axis accounting for 18.4%. Soil pH and TN had stronger effects on the soil bacterial community, factors explain a total of 64.6% of the variation in community composition (Fig. 7).

## Discussion

To reveal the bacterial diversity and community composition response to decreases and increases in precipitation in the Junggar desert, we examined five precipitation treatments. Using high throughput sequencing, we obtained 392,087 high quality sequences and examined the bacterial richness and diversity in each treatment. As expected, the results revealed significant differences in bacterial diversity between drought and increased precipitation treatment, but no significant differences in bacterial community composition among these treatments.

### The effects of precipitation on bacterial diversity

The soil microbial community plays a central role in the plant-soil feedback system (Prestel, Salamitou & Dubow, 2008). Therefore, the nature of the relationship between plant diversity and soil microbial diversity has been widely reported (Zak et al., 2003; Wang et al., 2015). For example, research has found that, the generally positive correlation between plant diversity and soil microbial diversity (Prober et al., 2015), will turn into a negative correlation when water and high levels of N are added (Zhang et al., 2018). In arid and semi-arid regions, increasing precipitation alleviated water limitations, and increased the number and abundance of dormant microorganisms to varying degrees, leading to an increase in the Simpson’s diversity index for soil bacteria (Tecon & Or, 2017). In our study, significant positive correlations of bacterial richness and diversity with plant richness and diversity were found where water was added (Tables 6 and 7). Millard & Singh (2010) suggested that increased plant diversity can promote productivity, and that higher productivity leads to greater litter decomposition and more root secretion, which, in turn, increases the soil organic carbon content. In consequence, the increase in plant diversity can result in an increase in soil humus concentration and improved nutrient conditions, providing better conditions for microbial growth (Lange et al., 2015; Wang et al., 2015).

In the present study, soil bacterial diversity and richness increased with increasing precipitation, and there were significant differences between the extreme drought treatment (−60%) and precipitation enhancement treatments (+30% and +60%) (Table 5). The reasons why the difference between precipitation manipulation treatments and the control treatment was not significant in the short term were that the numbers and abundance of species in the desert plant communities were relatively low, the coverage was low, the community species composition was relatively similar between the five treatments (Table 3), and it takes a long time for changes in plant diversity to affect soil properties. Li et al. (2018) suggested that extreme drought can significantly reduce the number and biomass of soil bacteria, because extreme drought soil conditions can inhibit soil microbial growth, and reduce the activity of the microbial community. In the present study, for example, we found that SOM and SWC under the extreme drought treatment were significantly lower than those under higher precipitation levels (Table 2). Furthermore, drought can indirectly affect soil microbial community characteristics by affecting plant physiological processes. For example, drought can restrict the flow of nutrients from the plant to the soil by affecting photosynthesis, which subsequently leads to a decline in soil microbial abundance and diversity (Rousk, Smith & Jones, 2013). It has also been found that drought can lead to a decrease in plant aboveground net primary productivity (ANPP), which leads to a decrease in the amount of carbon and nitrogen entering the soil, and ultimately to a decrease in soil microbial abundance (Beier et al., 2012).

### The effects of precipitation on bacterial community composition

Actinobacteria constitute one of the largest phyla among bacteria and represent Gram-positive bacteria, which can decompose considerable amounts of organic matter (Ventura et al., 2007). At the phylum level, Actinobacteria dominated changes in soil bacterial composition under extreme and moderate precipitation events (Zhang et al., 2019). Numerous studies have shown that Actinobacteria, Proteobacteria and Bacteroidetes are the main bacterial groups in desert soils, with the proportion of Actinomycetes reaching up to 50% (Bhatnagar & Bhatnagar, 2005; Bachar, Soares & Gillor, 2012). The abundance of the phylum Firmicutes, correlated negatively with soil moisture, being high in the deserts (Prestel, Salamitou & Dubow, 2008). Consistent with previous findings, at the phylum level, Actinobacteria, Proteobacteria and Bacteroidetes were dominated the composition of soil bacterial communities. In addition, Acidobacteria and Chloroflexi also account for a high proportion in the community (Fig. 6). Gram-positive Actinomycetes are tolerant of drought and can grow under dry conditions, and the relative abundance of Actinobacteria was higher in the less-watered treatments presumably because it has stronger cell walls, and can produce spores and grow in a filamentous pattern, which can effectively reduce the damage caused by drought and high temperatures (Koyama et al., 2018; Li et al., 2018). Proteobacteria and Bacteroidetes are widespread in desert soils, due to the presence of a variety of nutritional-type bacteria in these phyla (An et al., 2013).

There was little variation in the frequency patterns of these dominant components of soil bacterial communities under the different precipitation treatments in the present study, indicating that the dominant phyla of soil bacteria were stable under precipitation changes. The influence of precipitation change on soil bacterial community structure plays an important role in ecosystem function, such as decomposition of organic matter, transformation of nutrients, and maintenance of soil structure (Yuan et al., 2015). As the dominant components of soil bacterial communities did not change, it can be speculated that soil bacterial community has a regulatory mechanism to influence natural factors on a small scale, to ensure the stability of its composition and maintain its ecosystem function.

### The key factor driving the change of bacterial composition

Bacterial community composition can be correlated to an array of environmental factors (Fig. 7). Our studies indicated that indirect environmental factors, such as soil pH, that shift under precipitation changes, might play a larger role in determining microbial community composition than do direct changes to soil moisture. This finding is in accordance with earlier studies which showed that variation in bacterial community composition was strongly correlated with soil pH (Lauber et al., 2008; Yao et al., 2014). Fierer et al. (2009) revealed that soils in all biomes were dominated by the same soil bacterial phyla (Acidobacteria, Actinobacteria, Proteobacteria, and Bacteroidetes), with any variation in the composition of the bacterial communities being explained mostly by soil pH. The change in soil pH not only affects the diversity of soil bacteria directly (Fig. 5), but also affects it indirectly by affecting the supply of soil nutrients and the quantity and type of plant root exudates (Zhang et al., 2018). In addition, TN has an important effect on the structure and metabolism of bacterial community (Srinandan et al., 2012; Tu et al., 2019), and has a significant positive correlation with both the richness and biomass of microbial communities (Cordova-kreylos et al., 2006). In this research, we also found that soil pH and TN were the main factors affecting soil microbial community composition (Fig. 7), confirming that they were two of the most important factors involved in determining the composition of the soil microbial community. However, there were no significant differences in soil pH and TN among the five different precipitation treatments investigated in this study (Table 2), which may be the reason why there were no major differences in soil microbial community composition among the different precipitation treatments. Precipitation change is a long and gradual process, and many uncertainties remain to be resolved. Further field studies, with longer observation periods, will be necessary to disentangle the influences of precipitation changes on desert microbial communities.

## Conclusions

In the current study, we manipulated precipitation levels under field conditions to assess the response of soil bacterial diversity and community structure to precipitation changes. The results indicated that extreme drought significantly decreased soil bacterial diversity compared to increased precipitation, but the effects of increased precipitation on richness and diversity were not significant. There were no significant differences in bacterial community composition among the different precipitation treatments. The studies also indicated that bacterial diversity was positively correlated with plant diversity. Our findings demonstrated an inhibitory effect of extreme drought on soil microbial diversity in a short-term experiment and found the effect of increased precipitation on soil bacterial community was not significant. Precipitation change is a long and gradual process, and many uncertainties remain to be addressed, so that its cumulative effect on the components of the desert ecosystem will require long-term monitoring and research.

##  Supplemental Information

10.7717/peerj.8433/supp-1 Data S1 Raw data-alpha diversity of soil bacterial

10.7717/peerj.8433/supp-2 Data S2 Raw data-bacterial community composition

10.7717/peerj.8433/supp-3 Data S3 Raw data-plant diversity

10.7717/peerj.8433/supp-4 Data S4 Raw data-soil properties

## Additional Information and Declarations

### Data Availability

The following information was supplied regarding data availability: Data is available at NCBI SRA: SRR10020091–SRR10020105.
